# Comparative effects of nisin and monensin supplementation on growth performance, rumen fermentation, nutrient digestion, and plasma metabolites of fattening Hu sheep

**DOI:** 10.3389/fvets.2024.1441431

**Published:** 2024-08-01

**Authors:** Jiazhi Luo, Jun Jiang, Hongwei Duan, Han Zhang, Meijie Sun, Shengyong Mao, Junshi Shen

**Affiliations:** ^1^Laboratory of Gastrointestinal Microbiology, National Center for International Research on Animal Gut Nutrition, Nanjing Agricultural University, Nanjing, China; ^2^Ruminant Nutrition and Feed Engineering Technology Research Center, College of Animal Science and Technology, Nanjing Agricultural University, Nanjing, China

**Keywords:** nisin, fattening Hu sheep, growth performance, rumen fermentation, nutrient digestibility, monensin

## Abstract

**Introduction:**

This study was conducted to compare the effects of nisin (NIS) and ionophore antibiotic monensin (MON) on the growth performance, rumen fermentation, nutrient digestion and plasma metabolites of fattening Hu sheep.

**Methods:**

Thirty-six male Hu sheep (23.5 ± 1.0 kg) were divided into two blocks based on BW (low BW and high BW). Sheep within each block were then allotted to 9 pens respectively (two sheep/pen). Pens within each block were randomly assigned to one of three dietary treatments: (1) basal diet (CON); (2) basal diet + 40 mg/kg DM of MON; (3) basal diet + 274.5 mg/kg DM of NIS. The study lasted 9 weeks, with the initial 2 weeks for adaptation and the subsequent 7 weeks for treatment.

**Results:**

The results showed that both NIS and MON addition had no impacts on average daily gain (ADG), dry matter intake (DMI), and feed conservation rate (G:F) of sheep (*p* > 0.05). The digestibility of ether extract (EE) was lower in the MON-fed and NIS-fed sheep (*p* < 0.01) than in the CON group, whereas crude protein (CP) digestibility was higher in the MON-fed sheep compared to those fed NIS (*p* < 0.05). Both NIS and MON supplementation decreased acetate levels and acetate/propionate ratio in the rumen of Hu sheep (*p* < 0.05). Sheep fed MON exhibited higher total cholesterol concentrations (*p* < 0.05) compared to the CON and NIS groups. However, there were no significant differences in other plasma metabolites, including blood urea nitrogen (BUN), total bile acid, triglyceride, total protein, albumin, globulin, glucose, etc., among the three groups (*p* > 0.05).

**Discussion:**

In conclusion, dietary addition of NIS and MON altered the rumen fermentation mode by reducing acetate levels, with no discernible effects on the growth performance of the fattening Hu sheep.

## 1 Introduction

The increasing demand for livestock products such as milk, beef, and mutton has driven a shift toward large-scale and intensive livestock production, leading to higher livestock farming intensity and an elevated prevalence of diseases among livestock (1). Antibiotics are commonly utilized to enhance feed conservation rate and promote animal growth to meet societal demands for animal products and maximize economic benefits (2). In ruminant production, monensin (MON), the most widely employed antibiotic, is frequently used to modulate rumen fermentation, mitigate energy and nitrogen losses, and enhance feed conservation rate. Numerous studies have demonstrated that MON can optimize rumen fermentation patterns, suppress the production of methane, ammonia, and lactic acid in the rumen (3), and enhance the growth performance of ruminants (4). Nevertheless, the excessive use of antibiotics has led to the emergence of bacterial resistance and drug residue issues. In light of safety concerns, many countries have prohibited the use of antibiotics as dietary growth promoters for animals (5). Therefore, there is an urgent necessity to explore environmentally friendly, safe, effective, and residue-free alternatives to antibiotics to address this challenge.

Nisin (NIS), a 34-amino acid polypeptide antimicrobial substance, primarily inhibits gram-positive bacteria. It is the most extensively researched bacteriocin and has been approved by the FDA and the European Union as a food preservative (6, 7). Initially utilized in the food industry, NIS has demonstrated significant potential as an antibiotic alternative in recent years within the farming industry (8, 9). Prior studies have shown that NIS can enhance broiler growth performance through the modulation of gut microbiota composition, reduction of inflammatory responses, and intestinal apoptosis (7). Notably, Pogány Simonová et al. (8) observed a 9.4% increase in the average daily gain (ADG) of broiler rabbits fed NIS compared to a control group. Research in pigs has also indicated that NIS can influence the intestinal functional microbiota involved in acetate, butyrate, and propionate synthesis (10). Presently, most investigations on NIS in ruminants are conducted *in vitro* (11–13), and limited knowledge exists regarding its *in vivo* effects.

Previous *in vitro* studies have shown that both NIS and MON at appropriate concentrations can alter rumen fermentation patterns and increase propionate concentrations (11, 12). Unlike MON, NIS inhibited methane production without affecting dry matter disappearance rates (12). However, subsequent *in vivo* experiments, following the *in vitro* results, demonstrated that incorporating 30.5 mg/kg dry matter (DM) of NIS into the diet had no significant impact on the growth performance (14) and rumen microbiota of fattening Hu sheep (15). Notably, the *in vitro* artificial rumen lacks the complexity of the actual rumen environment in terms of substrates and microorganisms (16). It is hypothesized that the lack of the anticipated effects in previous animal experiments may be due to the insufficient dosage of NIS supplementation (14). Therefore, this study aimed to investigate the effects of a higher dose of NIS (274.5 mg/kg DM) on rumen fermentation and growth performance in fattening Hu sheep compared to MON.

## 2 Materials and methods

### 2.1 Animals, diets, and experimental design

The experimental procedures used in this study were approved by the Animal Care and Use Committee of Nanjing Agricultural University (protocol number: SYXK2017-0007).

Thirty-six healthy male Hu sheep (23.0 ± 1.0 kg) at the age of 3 months were divided into two blocks (18 sheep per block) based body weight (low BW: 22.3 ± 0.5 kg; high BW: 23.8 ± 0.6 kg). Within each block, the sheep were further divided into 9 pens (2 sheep/pen), and then randomly assigned to one of the three treatments: (1) basal diet (CON); (2) basal diet +40 mg/kg DM of MON; (3) basal diet +274.5 mg/kg DM of NIS. The NIS (Zhejiang New Yinxiang Bioengineering Co., Ltd.) used in this study contained 2.5% NIS with an activity of ≥1 × 10^6^ IU/g. The MON premix (Shandong Shengli Bioengineering Co., Ltd.) added to the MON treatment consisted of monensin sodium at a concentration of 20 g per 100 g of premix.

The basal diet was designed in accordance with the Feeding Standard of Meat Sheep (2004) issued by the Ministry of Agriculture of the People’s Republic of China (Table 1). The TMR was fed twice daily at 08:00 and 16:00 in all treatment groups, with a surplus of 5%–10% being ensured. All sheep were housed indoors pens (2 m × 4 m) with wooden slatted floors and had free access to drinking water. The study lasted for 9 weeks, incorporating a 2-week acclimatization phase followed by a 7-week experimental period.

**Table 1:** Composition and nutrient levels of basal diets (DM basis).

Item	Content
**Ingredient (% of DM)**	
Corn silage	23.00
Peanut vine	22.00
Corn grain	30.00
Soybean meal	7.00
DDGS	12.00
Wheat bran	2.00
Urea	0.75
NaCl	0.75
NaHCO_3_	1.00
CaCO_3_	1.00
CaHPO_4_	0.37
Mineral Premix^1^	0.10
Vitamin Premix^2^	0.03
**Nutrient composition, %**	
DM	49.7
CP	15.71
NDF	30.38
ADF	17.92
EE	3.51
Ash	9.48
DE, MJ/kg^3^	13.25

^1^Trace mineral element content per kg of premix: Cu 18,000 mg, Fe 35,500 mg, Co 300 mg, I 1,650 mg, Se 340 mg, Zn 42,000 mg, Mn 36,000 mg. ^2^Vitamin content per kg of premix: VA 20,040,000 IU, VD_3_ 6,600,000 IU, VE 200,000 IU. ^3^DE was a calculated value, while the others were measured values.

### 2.2 Sampling and measurement

The diet provided and refusals were measured daily during weeks 2, 4, 5, and 7 to calculate dry matter intake (DMI). Prior to morning feeding, the Hu sheep were weighed over 2 consecutive days in weeks 0, 4, and 7. These data were utilized to compute the average daily gain (ADG) of the sheep and the feed conservation rate (Gain:Feed, G:F), calculated as the ratio of ADG to DMI.

Ruminal fluid was obtained via an oral stomach tube around 3 h post morning feeding on the 5th day of weeks 4 and 7. The initial 50 mL of rumen fluid was discarded to minimize potential saliva contamination. Subsequently, the ruminal fluid was filtered through four layers of cheesecloth, and the pH of each sample was promptly measured using a portable pH-meter (Ecoscan pH-5, Eutech Instruments, Singapore). One milliliter of each ruminal fluid sample was preserved with the addition of 0.2 mL of 25% HPO_3_ for VFA analysis using gas chromatography (GC-2014AFsc, Shimadzu, Japan) as described by Shen et al. (12). Another 1 mL of each ruminal fluid sample was stored at −20°C for subsequent analysis for ammonia nitrogen using a colorimetric method (17).

Diet and ort samples were collected continually for 2 days in weeks 2, 4, 5, 7. The feeds were mixed thoroughly using the quadrat method and stored in a sealed container at −20°C. Over the final 3 days of the study, feces samples were collected twice daily and stored in sealed containers at −20°C. The feed samples from each group and fecal samples from each sheep were combined for three consecutive days, subsequently dried in an oven for 48 h at 65°C. The dried samples were pulverized through a 1-mm screen utilizing a Cyclotec mill (Tecator 1093; Tecator AB, Höganäs, Sweden). Subsequently, all samples underwent analysis for dry matter (DM) (18), organic matter (OM) (18), and crude protein (CP) (18). The contents of ADF and NDF were determined according to Van Soest et al. (19). The acid-insoluble ash (AIA) in both the diet and fecal samples was measured according to the procedure outlined by Van Keulen and Young (20), which served as internal markers for estimating apparent nutrient digestibility.

On the 6th day of weeks 4 and 7 at approximately 3 h after morning feeding, blood samples were drawn from the jugular vein of each sheep into blood collection tube containing sodium heparin. The sample was then centrifuged at 3,000 × g/min for 10 min to separate plasma. Subsequently, all plasma samples were stored at −20°C for subsequent analysis of blood urea nitrogen (BUN), glucose, total protein (TP), albumin, creatinine (CRE), total cholesterol (TCHO), triglyceride (TG), total bile acid (TBA), alkaline phosphatase (ALP), aspartate aminotransferase (AST) and alanine aminotransferase (ALT) using commercial kits (Nanjing Jiancheng Technology Co., Ltd.). The Globulin levels were calculated by determining the difference between TP and albumin.

### 2.3 Statistical analyses

The statistical analyses were performed using the mixed model procedure of SAS version 9.4. The observational unit was defined as the pen when measurements were taken collectively for all aspects related to the pen, including DMI, ADG, feed conservation rate, nutrient intake, and apparent digestibility. Conversely, the observational unit was considered as the individual sheep when measurements were conducted on each lamb within the pen for parameters like BW, rumen fermentation characteristics, and plasma metabolites. Data for DMI, ADG, feed conservation rate, rumen fermentation characteristics, and plasma metabolites were analyzed with week as repeated measures. The statistical models incorporated fixed effects of treatment and week, together with their interaction, along with random effects of block, pen × block × treatment, and lambs within the pen × block × treatment. In contrast, data for BW, nutrients intake and apparent digestibility, the statistical models comprised fixed effects of treatment, along with random effects of block, pen × block × treatment, and lambs within the pen × block × treatment. When the pen served as the observational unit, the random effects in the aforementioned models were simplified to block and pen × block × treatment. Degrees of freedom were determined utilizing the Kenward-Roger option. Differences were considered to be statistically significant when the *p*-values were ≤ 0.05, and trends were declared at 0.05 < *p* ≤ 0.10. All reported values are least squares means unless otherwise stated.

## 3 Results

### 3.1 Growth performance

No difference (*p* > 0.05) in initial BW were observed across all treatments (Table 2). Dietary NIS and MON addition had no effect (*p* > 0.05) on the final BW, ADG, DMI and feed conservation rate of fattening Hu sheep. Nonetheless, the ADG of 267.8 g/d was the highest among fattening Hu sheep fed with MON compared to the other two groups.

**Table 2:** Comparative effects of nisin and monensin supplementations on growth performance of fattening Hu sheep.

Item	CON	MON	NIS	SEM	*p*-value		
					Trt	Wk	Trt × Wk
BW, kg					-	-	-
Initial	22.92	23.01	23.17	0.792	0.54	-	-
Final	35.76	36.23	35.75	0.796	0.74	-	-
DMI, g/d	1194.9	1178.5	1180.8	46.88	0.95	<0.01	0.76
ADG, g/d	260.8	267.8	255.3	9.30	0.65	<0.01	0.79
G:F	0.22	0.23	0.22	0.006	0.37	<0.01	0.40

CON, control (no additives); MON, monensin, 40 mg/kg DM; NIS, nisin, 274.5 mg/kg DM; Trt, treatment; Wk, week; Trt × Wk, interaction of treatment and week; G:F, Gain:Feed.

### 3.2 Rumen fermentation characteristics

The inclusion of dietary NIS and MON resulted in a significant decrease (*p* < 0.05) in the concentration of acetate and the acetate/propionate ratio in the rumen of the fattening Hu sheep (Table 3), accompanied by a tendency toward decreased butyrate concentrations (*p* = 0.07). Notably, the NIS group exhibited significantly lower isobutyrate concentrations compared to the other treatment groups (*p* < 0.05). However, the dietary addition of NIS and MON had no impact on rumen pH, NH_3_-N, TVFA, propionate, valerate, isovalerate and TBCFA concentrations in fattening Hu sheep (*p* > 0.05).

**Table 3:** Comparative effects of nisin and monensin supplementations on ruminal fermentation of fattening Hu sheep.

Item	CON	MON	NIS	SEM	*p*-value		
					Trt	Wk	Trt × Wk
pH	6.37	6.46	6.52	0.079	0.20	0.49	0.89
NH_3_-N, mg/dL	13.82	11.79	14.43	1.955	0.52	0.06	0.07
Total VFA, mM	97.9	90.8	88.6	3.27	0.16	0.83	0.86
Acetate, mM	62.0^a^	55.4^b^	54.8^b^	2.03	0.05	0.92	0.88
Propionate, mM	20.9	22.2	20.6	0.94	0.47	0.95	0.64
A:P	2.99^a^	2.52^b^	2.70^b^	0.082	<0.01	0.62	0.78
Butyrate, mM	11.91	10.09	10.27	0.532	0.07	0.14	0.35
Valerate, mM	1.08	0.95	0.94	0.059	0.21	0.97	0.89
Isobutyrate, mM	0.77^a^	0.77^a^	0.65^b^	0.036	0.04	0.35	0.88
Isovalerate, mM	1.21	1.45	1.26	0.096	0.24	0.06	0.86
TBCVFA, mM	1.98	2.22	1.91	0.126	0.22	0.27	0.86

CON, control (no additives); MON, monensin, 40 mg/kg DM; NIS, nisin, 274.5 mg/kg DM; Trt, treatment; Wk, week; Trt × Wk, interaction of treatment and week; A:P, acetate:propionate; TBCVFA, total branched-chain VFA. ^a,b^Means within a row with different superscripts differ (*p* < 0.05).

### 3.3 Nutrients intake and apparent digestibility

Dietary NIS and MON addition had no effect (*p* > 0.05) on the intake of DM, OM, CP, NDF, ADF and EE in fattening Hu sheep (Table 4). Both NIS and MON groups notably reduced the digestibility of EE (*p* < 0.05) in comparison to the CON group (Table 4). Furthermore, the MON group exhibited significantly higher digestibility of CP in contrast to the NIS group (*p* < 0.05). However, there were no differences in the digestibility of DM, OM, NDF and ADF among the treatments (*p* > 0.05).

**Table 4:** Comparative effects of nisin and monensin supplementations on nutrient digestion of fattening Hu sheep.

Item	CON	MON	NIS	SEM	*p*-value
**Intake, g/d**					
DM	1302.5	1322.8	1289.9	90.5	0.92
OM	1179.3	1197.3	1167.8	81.91	0.92
CP	203.6	208.4	200.6	14.12	0.83
NDF	392.1	398.6	393.0	27.37	0.96
ADF	228.6	235.4	234.7	16.14	0.88
EE	45.6	46.2	45.1	3.16	0.93
**Digestibility, %**					
DM	72.7	73.0	72.5	0.80	0.93
OM	77.8	78.3	77.6	0.71	0.79
CP	73.9^ab^	75.3^a^	73.2^b^	0.51	0.03
NDF	53.8	55.7	52.0	1.66	0.31
ADF	52.5	54.1	53.2	1.84	0.84
EE	84.9^a^	80.0^b^	78.0^b^	1.25	<0.01

CON, control (no additives); MON, monensin, 40 mg/kg DM; NIS, nisin, 274.5 mg/kg DM. ^a,b^Means within a row with different superscripts differ (*P* < 0.05).

### 3.4 Plasma metabolites

Compared with the CON and NIS groups, MON addition significantly elevated the blood levels of TCHO in Hu sheep (*p* < 0.05; Table 5). However, the addition of NIS and MON in the diet had no effect (*p* > 0.05) on the plasma levels of ALP, ALT, AST, BUN, CRE, glucose, TBA, TG, TP, albumin, and globulin in fattening Hu sheep (Table 5).

**Table 5:** Comparative effects of nisin and monensin supplementations on blood biochemical parameters of fattening Hu sheep.

Item	CON	MON	NIS	SEM	*p*-value		
					Trt	Wk	Trt × Wk
ALP, U/L	443.0	457.4	433.5	34.14	0.88	0.16	0.93
ALT, U/L	15.4	13.7	13.1	1.16	0.38	<0.01	0.56
AST, U/L	119.1	121.4	114.0	6.59	0.53	0.30	0.06
BUN, mg/dL	28.0	28.9	28.1	0.94	0.58	0.64	0.04
CRE, mg/dL	0.71	0.71	0.72	0.029	0.96	0.80	0.67
Glucose, mg/dL	64.5	66.0	65.7	1.35	0.50	<0.01	<0.01
TBA, μmol/L	10.61	10.32	12.70	1.279	0.37	0.31	0.86
TCHO, mg/dL	37.1^b^	45.2^a^	37.3^b^	2.15	<0.01	<0.01	0.09
TG, mg/dL	33.5	34.5	36.2	0.95	0.14	0.28	0.46
TP, g/dL	4.88	4.81	4.83	0.104	0.83	0.97	0.06
Albumin, g/dL	1.92	2.00	1.96	0.024	0.10	<0.01	0.01
Globulin, g/dL	2.95	2.81	2.87	0.076	0.42	0.37	0.37
A:G	0.66	0.72	0.69	0.019	0.08	0.06	0.61

CON, control (no additives); MON, monensin, 40 mg/kg DM; NIS, nisin, 274.5 mg/kg DM; Trt, treatment; Wk, week; Trt × Wk, interaction of treatment and week; ALP, alkaline phosphatase; ALT, alanine aminotransferase; AST, aspartate aminotransferase; BUN, blood urea nitrogen; CRE, creatinine; TBA, total bile acid; TCHO, total cholesterol; TG, triglyceride; TP, total protein; A:G, Albumin:Globulin. ^a,b^Means within a row with different superscripts differ (*P* < 0.05).

## 4 Discussion

### 4.1 Growth performance

MON is a commonly used antimicrobial growth promoter in livestock production aimed at enhancing feed conservation rate. The mechanism by which MON improves energy efficiency is primarily linked to increased ruminal propionate synthesis (21). Interestingly, in the current study, it was observed that dietary supplementation of MON had no impact on ADG and feed conservation rate in Hu sheep, which corresponded to the absence of changes in propionate concentration. The efficacy of MON on feed conservation rate is influenced by various factors, such as dosage, feeding strategies, and the dietary nutrient composition. Vyas et al. (22) reported that incorporating 33 mg/kg DM of MON into a high-forage (65%) diet led to enhanced feed conservation rate, whereas its addition to a high-concentrate (92%) diet showed no effect on feed conservation rate. This discrepancy can be attributed to the higher energy content in high-grain diets, which stimulates a relatively higher production of ruminal propionate compared to high-forage diets (23). Consequently, the supplementation of MON in high-concentrate diets had no noticeable effect on feed conservation rate. In the current study, where the dietary composition was balanced (forage:concentrate = 45:55) to meet the energy requirements of Hu sheep, it is plausible that the feed conservation rate remained unaffected. Correspondingly, NIS exhibited a parallel effect to MON, consistent with the results of a previous study by Shen et al. (14), where supplementation with 30.5 mg/kg DM of NIS yielded similar results. The influence of NIS observed in this study may be influenced by the dietary forage-to-concentrate ratio similarly to MON, potentially limiting its ability to significantly enhance growth.

### 4.2 Rumen fermentation characteristics

In the present study, it was found that adding 274.5 mg/kg DM of NIS and 40 mg/kg DM of MON to the diet decreased acetate concentration and altered rumen fermentation pattern, which is consistent with the results of previous studies (12, 24). Acetate is primarily produced through the microbial fermentation of fibrous materials. It has been reported that both NIS and MON can inhibit acetate production by increasing the permeability of cell membranes to suppress gram-positive fiber-degrading bacteria in the rumen, such as Ruminococcus spp. and other major acetate-producing bacteria (12). The reduced acetate concentration in the present study indicated that the addition of NIS and MON may also inhibit the abundance of cellulolytic bacteria in Hu sheep. Further studies are warranted to assess the impact of NIS and MON on ruminal microorganisms.

Following the decrease in the acetate/propionate ratio, a shift in rumen fermentation mode is indicated. The VFA profile is closely related to the hydrogen metabolism pathway and methane production in the rumen. Acetate and butyrate formation releases hydrogen, aiding methane synthesis, whereas propionate formation competes for hydrogen utilization in the rumen. Although methane production was not quantified in the present study, an estimation based on the equation proposed by Moss et al. (25) indicated a reduction in methane production with NIS supplementation. Previous studies have shown that dietary NIS supplementation can inhibit rumen methane production (12, 13, 26). Therefore, direct measurement of methane production is advisable for validation. Furthermore, it is generally believed that inhibiting methane production can alter hydrogen utilization pathways, reduce energy loss and improve production performance (27). However, the present study did not find an improvement in ADG or feed conservation rate in fattening Hu sheep, aligning with the findings of a previous low-dose *in vivo* study (14). This suggests that the conserved energy is not digested, absorbed, and utilized effectively by the host.

Ruminal propionate is the principal substrate for hepatic gluconeogenesis, where glucose synthesis from propionate significantly contributes to the total energy production in ruminants, ranging from 24% to 61% (28). In the current study, the ruminal concentration of propionate in Hu sheep fed with NIS and MON did not exhibit significant variations. The results are in agreement with those of Santoso et al. (29) and Benchaar et al. (30), but inconsistent with previous reports that NIS and MON can enhance propionate production (12, 31). Several pathways, such as the succinate pathway, acrylate pathway, and tricarboxylic acid cycle, are involved in ruminal propionate synthesis (32). Shen et al. (12) reported that the addition of 1 μM and 5 μM nisin led to an increase in the relative abundance of genera with succinate as the metabolic end product, such as *Fibrobacter succinogenes* and Succinivibrio, and increased the concentration of propionate through the succinate pathway. It also reduces the relative abundance of streptococci involved in the acrylate pathway, thereby weakening the production of propionate through the acrylate pathway. Therefore, the compensatory interactions between these metabolic pathways may explain the stable propionate levels observed in this study. Additionally, dietary composition, particularly the concentrate-to-forage ratio, plays a significant role in the effectiveness of additives. Ramanzin et al. (33) observed a more pronounced effect of MON on propionate concentration in diets with lower forage content (50%) compared to those with higher forage content (70%). Hence, the discrepancies in study results may be due to variations in dosage, dietary composition etc.

### 4.3 Nutrients intake and apparent digestibility

Shen et al. (14) previously reported that dietary supplementation with 30.5 mg/kg DM of NIS had no effect on the digestibility of DM, NDF, ADF, EE and CP in fattening sheep, which consistent with the results of the present study. This similarity in outcomes may be attributed to the maintenance or potential increase in the population of rumen protozoa and the relative abundance of major cellulolytic bacteria, as suggested by Shen et al. (12). However, Azzaz et al. (34) found that adding 500 U/kg NIS improved nutrient digestibility in ewes. Discrepancies in results observed across studies could be influenced by factors such as species differences, gender, and nutritional composition. Furthermore, in the current study, the inclusion of NIS significantly decreased the digestibility of EE. The rationale behind this finding remains unclear. Conducting further analysis on intestinal enzyme activity could potentially provide insights into the observed variations in EE digestibility.

This study found that dietary supplementation of MON had no effect on DM digestibility, which was consistent with previous study in cannulated wethers (35). In contrast, Soltan et al. (36) found that the addition of monensin led to reduced DM digestibility. The adverse effect of MON on feed digestion is primarily attributed to its inhibition of cellulolytic bacteria, such as *Fibrobacter succinogenes*. However, research has indicated that *Fibrobacter succinogenes* can develop resistance to MON over time. Thus potentially explaining the lack of effect on DM digestibility with MON supplementation (11, 37). Moreover, in the present study, the CP digestibility of the MON group exceeded that of the NIS group, consistent with the findings of Polizel et al. (35). MON may reduce MCP synthesis by decreasing the population of rumen bacteria, subsequently diminishing the proportion of RDP and elevating RUP levels (38). Studies have shown that RUP is more readily digested in the small intestine compared to microbial proteins (39), and the addition of MON can enhance amino acid absorption in the small intestine (40). Therefore, the increased CP digestibility in the MON group may be associated with heightened CP digestion and absorption rates in the small intestine.

### 4.4 Plasma metabolites

Blood metabolites are crucial indicators of the nutritional and health status of animals. In this study, the plasma metabolites of Hu sheep in each treatment were found to be within the normal range (41), suggesting that the dietary inclusion of NIS and MON did not have any adverse effects on the metabolism or health of the fattening Hu sheep. Shen et al. (14) reported that dietary supplementation of 30.5 mg/kg DM of NIS had no significant impact on the blood biochemical parameters of fattening sheep, consistent with our findings. Furthermore, previous studies have indicated that the levels of ALT, ALP, and AST in the blood are linked to inflammation, while the concentrations of globulin and albumin can serve as indicators of humoral immunity and protein synthesis in animals (42). These outcomes indirectly imply that the incorporation of MON and NIS into the diet does not affect the immune response of the Hu sheep.

The current study noted an elevation in the blood concentration of total cholesterol in Hu sheep supplemented with MON. Comparable findings have been documented by Duffield et al. (43) and O’Kelly and Spiers (44). It has been reported that MON can enhance energy metabolism by promoting fat export from the liver while decreasing the fat transported to the liver and reducing non-esterified fatty acids (NEFA), consequently mitigating liver fat accumulation and enhancing liver function (43). It is recognized that hepatic fat export is frequently released into the blood as lipoproteins, composed of triacylglycerol, cholesterol, and phospholipids synthesized in the liver with apolipoproteins. Hence, the rise in cholesterol levels triggered by MON supplementation may result from increased lipoprotein export from the liver. Further studies investigating the mechanisms of energy metabolism are needed to validate this hypothesis.

## 5 Conclusion

Both dietary supplements of NIS and MON reduced rumen acetate concentration and induced changes in the rumen fermentation pattern. However, neither of these additives impacted the growth performance or health status of the fattening Hu sheep. The regulatory mechanism of NIS and MON on rumen fermentation needs further investigation, especially through microbiome analysis. Moreover, the inclusion of MON in the diet led to elevated total cholesterol levels in the blood of Hu sheep in comparison to the other groups. The variations in cholesterol levels may be linked to energy metabolism, warranting further research on the specific metabolic pathways.

## Data availability statement

The original contributions presented in the study are included in the article/supplementary material, further inquiries can be directed to the corresponding author.

## Ethics statement

All animal protocols were approved by the Animal Care and Use Committee of Nanjing Agricultural University (protocol number: SYXK2017-0007). The studies were conducted in accordance with the local legislation and institutional requirements. Written informed consent was obtained from the owners for the participation of their animals in this study.

## Author contributions

JL: Formal analysis, Writing – original draft. JJ: Writing – original draft, Formal analysis. HD: Writing – review & editing. HZ: Writing – review & editing. MS: Methodology, Writing – review & editing. SM: Resources, Writing – review & editing. JS: Conceptualization, Formal analysis, Funding acquisition, Supervision, Writing – review & editing.
